# Early Life Febrile Seizures Impair Hippocampal Synaptic Plasticity in Young Rats

**DOI:** 10.3390/ijms22158218

**Published:** 2021-07-30

**Authors:** Tatyana Y. Postnikova, Alexandra V. Griflyuk, Dmitry V. Amakhin, Anna A. Kovalenko, Elena B. Soboleva, Olga E. Zubareva, Aleksey V. Zaitsev

**Affiliations:** Sechenov Institute of Evolutionary Physiology and Biochemistry of RAS, 44, Toreza Prospekt, 194223 Saint Petersburg, Russia; tapost2@mail.ru (T.Y.P.); griflyuk.al@mail.ru (A.V.G.); dmitry.amakhin@gmail.com (D.V.A.); kovalenko_0911@mail.ru (A.A.K.); soboleva.elena.1707@gmail.com (E.B.S.); zubarevaoe@mail.ru (O.E.Z.)

**Keywords:** febrile seizures, hyperthermia, long-term potentiation, NMDA receptor, hippocampus

## Abstract

Febrile seizures (FSs) in early life are significant risk factors of neurological disorders and cognitive impairment in later life. However, existing data about the impact of FSs on the developing brain are conflicting. We aimed to investigate morphological and functional changes in the hippocampus of young rats exposed to hyperthermia-induced seizures at postnatal day 10. We found that FSs led to a slight morphological disturbance. The cell numbers decreased by 10% in the CA1 and hilus but did not reduce in the CA3 or dentate gyrus areas. In contrast, functional impairments were robust. Long-term potentiation (LTP) in CA3-CA1 synapses was strongly reduced, which we attribute to the insufficient activity of N-methyl-D-aspartate receptors (NMDARs). Using whole-cell recordings, we found higher desensitization of NMDAR currents in the FS group. Since the desensitization of NMDARs depends on subunit composition, we analyzed NMDAR current decays and gene expression of subunits, which revealed no differences between control and FS rats. We suggest that an increased desensitization is due to insufficient activation of the glycine site of NMDARs, as the application of D-serine, the glycine site agonist, allows the restoration of LTP to a control value. Our results reveal a new molecular mechanism of FS impact on the developing brain.

## 1. Introduction

Infectious diseases with fever can provoke febrile seizures (FSs) [1,2], which constitute one of the most common neurological disorders in children between 3 months and 5 years [3]. FSs are divided into simple FSs (lasting < 10–15 min) and complex FSs (lasting >15 min) [4,5]. This classification is currently accepted as having predictive value. In the vast majority, simple FSs are benign and do not have adverse long-term effects on children’s development [6]. Clinical studies have shown that adults who experienced more prolonged FSs in early life frequently demonstrate a broad range of neurological disorders in later life [7,8,9]. Retrospective analyses of adults with temporal lobe epilepsy identified a high prevalence (30–50%) of a history of prolonged FSs during early childhood [5]. As with many other negative factors acting in early life, the FSs can also cause long-lasting alteration of cognitive functions, particularly learning and memory [10,11,12,13,14].

To study the mechanisms underlying the impact of FSs on the developing brain, a model of hyperthermia-induced seizures in neonatal rats was established [7,15,16]. Epileptic seizures strongly affect glutamatergic transmission [17,18]. Since the glutamatergic transmission is critically involved in learning and memory, it is not surprising that early seizures lead to cognitive impairment.

It was shown that prolonged experimental FSs induce transient hippocampal neuronal injury [19,20], leading to long-lasting alterations in properties of hippocampal neurons, with profound consequences on the excitability of the hippocampal network [21]. These and other alterations enhanced rats’ susceptibility to further limbic seizures throughout life [22] and induce recurrent, spontaneous seizures later in life in 35% of animals [23].

Experimental data on the effect of FSs on synaptic plasticity have been described in a few studies and are contradictory. Chang et al. (2005) found that repeated FSs lead to the long-term bidirectional modulation of synaptic plasticity in the CA1 region of the hippocampus, specifically, the attenuation of long-term potentiation (LTP) and facilitation of long-term synaptic depression (LTD) [24]. However, Notenboom et al. (2010) demonstrated an increase in LTP and decreased LTD in rats at P44 following FSs at P10 [25].

The long-term impairment of synaptic plasticity and cognitive function in rodents has also been observed in other models of induced neonatal seizures [26,27,28,29,30,31,32]. For example, in a model of neonatal seizures induced by hypoxia on P10, an increase in LTP was observed immediately after the seizure [33]. The attenuation of LTP was recorded 48–72 h after the seizures, which persisted in the rats at adulthood (P60) [34]. However, the precise mechanisms that cause changes in hippocampal plasticity following early life seizures have remained obscure.

In the present study, we aimed to investigate the morphological and functional changes in the hippocampus of young rats exposed to FSs, in order to better understand the mechanisms of plasticity impairment. Only animals with prolonged FSs that lasted at least 15 min were included in the experiments. The study was performed 11 days after FSs.

## 2. Results

### 2.1. Febrile Seizures Led to a Decrease in the Number of Neurons in the CA1 Area and Hilus of the Hippocampus

Whether FSs induce neuronal cell death has remained controversial. Prospective and retrospective clinical studies suggest that prolonged FSs may lead to neuronal cell death and mesial temporal sclerosis [5,7,8,11,35]. Animal studies indicate that experimental FSs in early life may lead to sustained dysfunction of hippocampal neurons without cell death [19,20].

**Figure 1:**
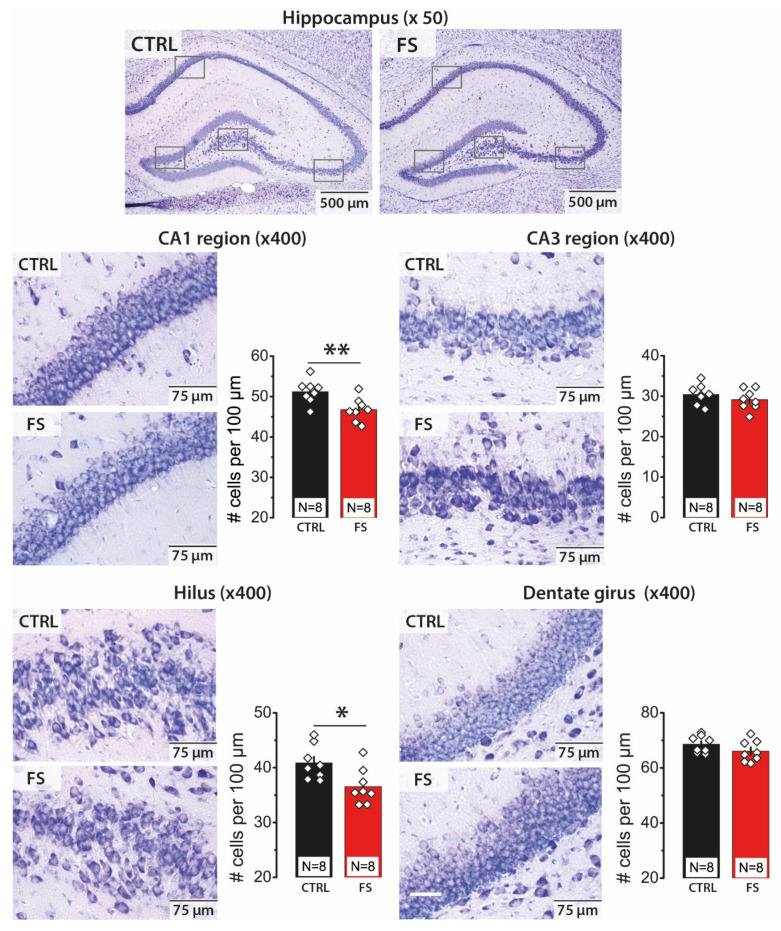
Nissl staining of the neurons in control (CTRL) and FS rats (FS) in the hippocampal areas CA1, CA3, hilus and dentate gyrus (DG). Group data of the counted Nissl stained neurons per 100 μm of the cellular layer. The rhombuses show individual values per brain. The bars indicate average values, and error bars show standard errors of the means. * *p* < 0.05, ** *p* < 0.01, *t*-test.

Using Nissl staining (Figure 1), we counted neurons in pyramidal layers of CA1 and CA3 areas of the hippocampus, hilus and granular cell layer of the DG. We detected a decline in cell numbers in CA1 (51.2 ± 1.0 cells per 100 μm in control, N = 8 rats, vs. 46.8 ± 1.0 cells per 100 μm after FS, N = 8 rats; *t*-test = 3.07, *p* < 0.01) and hilus (40.9 ± 1.1 cells in control, vs. 36.6 ± 1.1 cells after FS; t = 2.72, *p* < 0.05). No significant changes in the number of neurons were observed in CA3 (30.4 ± 0.9 cells in control, vs. 29.1 ± 0.9 cells after FS; t = 1.05, *p* = 0.31) and DG (68.6 ± 1.1 cells in control; 66.1 ± 1.4 cells after FS; t = 1.44, *p* = 0.17).

Thus, we demonstrated that FS leads to as much as a 10% decrease in cell numbers in some hippocampal areas. CA1 and the hilus are the areas most vulnerable to FSs, while no apparent changes in the number of cells in CA3 and DG were detected. Since information processing in the hippocampus was determined by excitatory synaptic connections within and between regions; changes in the ratio of the number of neurons in the different hippocampal areas can lead to some functional disturbances in the hippocampus.

### 2.2. The Efficacy of Synaptic Neurotransmission at CA3-CA1 Is Reduced after FS

Next, we investigated whether the efficacy of synaptic neurotransmission at CA3-CA1 pyramidal neuron synapses changes following FS. Afferent fibers from the CA3 area were electrically stimulated at the CA2/CA1 border using tungsten bipolar electrodes. We applied currents in a range of intensities from 25 to 300 mA and measured amplitudes of presynaptic FVs, which indicated the number of CA3 axons that fired action potentials and fEPSPs, showing the overall excitatory postsynaptic response occurring in CA1 neurons.

**Figure 2:**
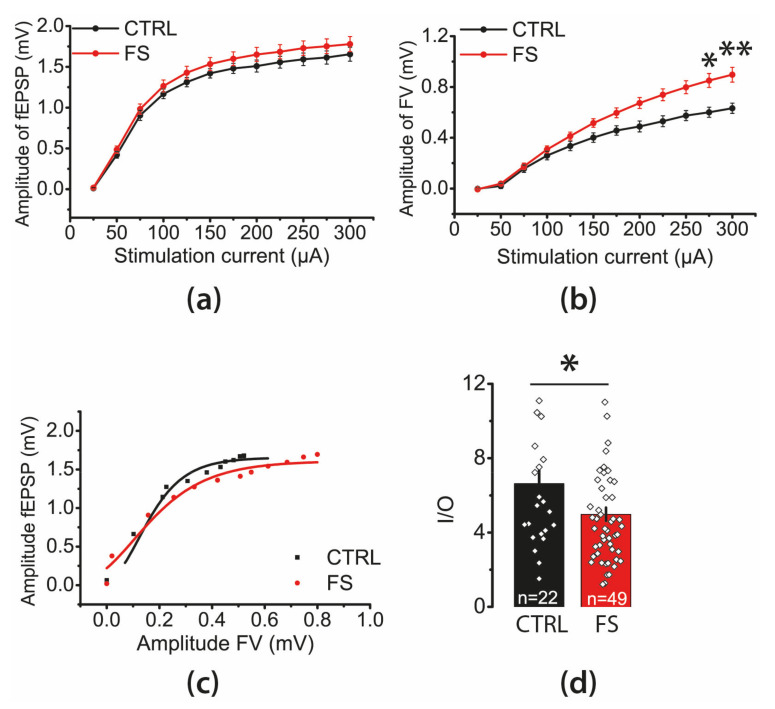
Stimulation–response relationships for fEPSP amplitudes (**a**) and presynaptic FV amplitudes (**b**) recorded from the hippocampal CA1 area in control (CTRL) and FS rats. Each point represents the mean ± SEM. (**c**) Representative examples of I/O relationships between the fEPSP and FV amplitudes in hippocampal slices of control and FS rats. (**d**) The maximal I/O slopes were smaller in FS rats than in control animals. Each rhombus represents a value obtained in individual brain slice. One to three slices were used per animal. * *p* < 0.05, ** *p* < 0.01—the difference between control and FS groups.

First, we compared the relationships between the amplitudes of fEPSPs and current intensity in control (N = 12 animals, *n* = 27 slices) and FS animals (N = 23 rats, *n* = 53 slices); no differences were found between the groups (repeated measures ANOVA F_11,858_ = 0.50; *p* = 0.90, Figure 2a). In contrast, there was a significant increase in FVs amplitudes following FS (F_11,836_ = 8.7; *p* < 0.001, control: *n* = 26, FS: *n* = 52; Figure 2b), suggesting a higher excitability of presynaptic fibers.

Next, we compared the neuronal input–output (I/O) relationships between the fEPSP and FV amplitudes in the control and FS groups. The maximal I/O slope in such a curve is considered a synaptic strength measure [36]. We determined the maximal I/O slope for each slice by fitting experimental data with a sigmoidal Gompertz function (Figure 2c) and noticed that the average slope was reduced by 25% in post-FS rats (*t*-test: t = 2.30; *p* < 0.05; control: *n* = 22, FS: *n* = 49; Figure 2d). These data indicate that the synaptic neurotransmission efficacy at CA3–CA1 was reduced after FS.

### 2.3. Short-Term Synaptic Plasticity of Hippocampal Neurons Does not Change in Rats that Underwent FS in Early Postnatal Ontogenesis

**Figure 3:**
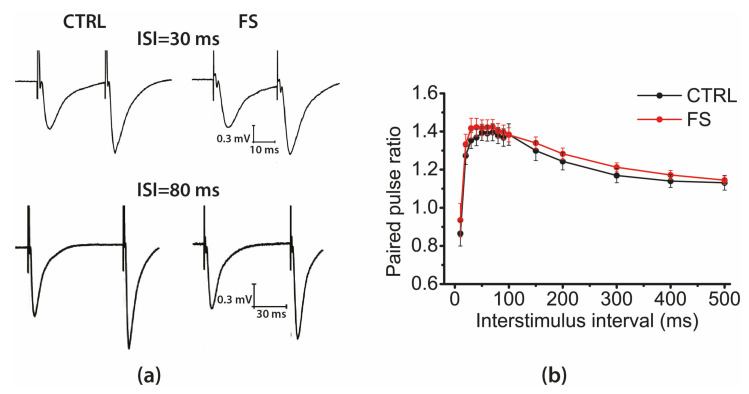
Short-term synaptic plasticity did not change in rat hippocampal slices after FS. (**a**) Representative examples of pair-pulse responses from the hippocampal CA1 area in control (CTRL) and post-FS rats (FS), interpulse intervals of 30 ms and 80 ms. (**b**) Diagram showing the paired-pulse facilitation in hippocampal slices across different inter-stimuli. Each point represents the mean ± SEM.

The disruption in synaptic transmission efficacy may be due to disturbances of presynaptic mechanisms. As a first approximation, changes in the probability of mediator release can be determined by changes in short-term synaptic plasticity properties [37]. We used a paired-pulse stimulation to study the possible changes in short-term synaptic plasticity and compared fEPSP amplitude ratios at different interstimulus intervals, ranging from 10 to 500 ms in animals of the control (N = 7 rats, *n* = 12 slices) and experimental (N = 9, *n* = 15) groups. Figure 3 shows examples of the paired fEPSP at the interstimulus intervals of 30 ms and 80 ms, and the curves illustrating the dependence of the paired amplitude ratios on the interstimulus intervals’ values. Repeated measures ANOVA revealed no significant effect of FS (F_1,336_ = 0.20; *p* = 0.66) or interaction of factors (interstimulus interval duration × FS; F_14,336_ = 0.26; *p* = 0.99) on the magnitude of short-term facilitation.

Thus, the absence of differences in short-term plasticity between the control and experimental groups suggests that the probability of neurotransmitter release in the CA1 field does not change after FS.

### 2.4. Febrile Seizures Impair Long-Term Synaptic Plasticity and Synaptic Transmission in the Rat Hippocampus

**Figure 4:**
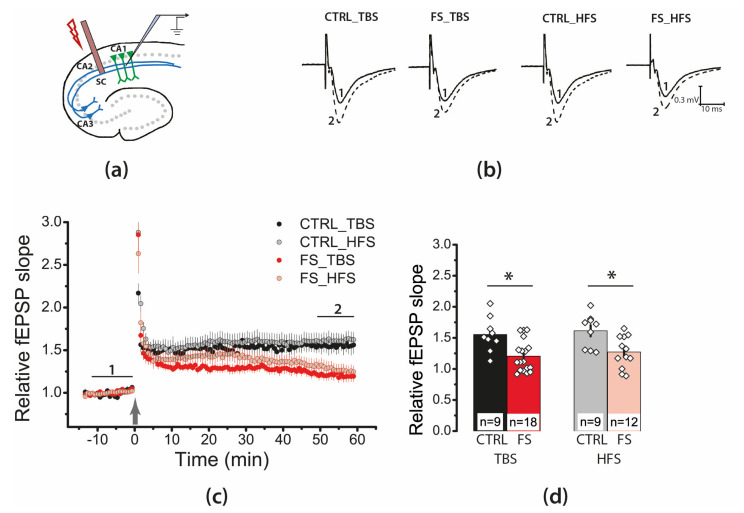
Long-term synaptic potentiation (LTP) was weakened in the CA1 of juvenile rats’ hippocampus after early life FSs. (**a**) Schema showing the positions of electrodes in the hippocampus. (**b**) Representative examples of fEPSP before induction (1) and 60 min after HFS or TBS (2). (**c**) Diagram showing changes in the value of the normalized slope of fEPSP in control (CTRL) and experimental (FS) animals after theta-burst stimulation (TBS) or after high-frequency stimulation (HFS) (stimulation was carried out at time 0). (**d**) Diagram illustrates the differences in LTP between control (CTRL) and experimental (FS) animals with different stimulation types. All data in this and the following figures are presented as a mean ± standard error of the mean. * *p* < 0.05—the significant difference with the control group (Tukey’s post hoc test).

Next, we investigated whether LTP properties at the CA3–CA1 synapses of the hippocampus change after FSs. We applied two LTP induction protocols: a theta-burst stimulation (TBS) and high-frequency stimulation (HFS). Both protocols were less effective for LTP induction in the FS group (Figure 4). According to a two-way ANOVA, FSs significantly attenuated LTP (FS/control, F_1,44_ = 19.1, *p* < 0.001), regardless of the type of protocol used (protocol type × FS/control, F_1,44_ = 0.001, *p* = 0.98). The TBS protocol induced an LTP of 1.55 ± 0.09 in the control group (N = 6 rats, *n* = 9 slices), while only 1.21 ± 0.06 in the FS group (N = 8, *n* = 18), HFS protocol—1.61 ± 0.08 (N = 7, *n* = 9) and 1.27 ± 0.07 (N = 6, *n* = 12), respectively.

Thus, a significant weakening of LTP in the CA1 hippocampal field of juvenile rats was observed following FS.

**Figure 5:**
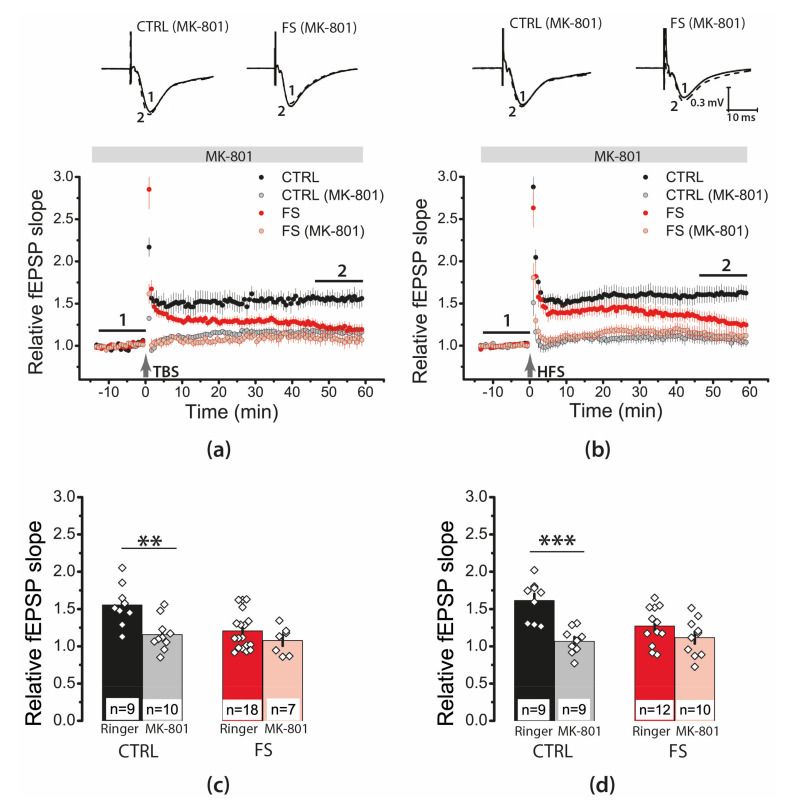
LTP induction in the CA1 hippocampus of juvenile animals after early life FS was NMDAR-dependent. The normalized fEPSP slope in the control and experimental groups in the presence of the NMDAR blocker MK-801 (10 μM), before and after TBS (**a**) or HFS (**b**). (**c**,**d**) Diagrams illustrating the magnitude of plasticity in the control and experimental groups in the presence of MK-801, after TBS (**c**) or HFS (**d**). Note that no synaptic plasticity was detected in the presence of MK-801 in any group. Two-way ANOVA following Tukey post hoc tests were used. ** *p* < 0.01, *** *p* < 0.001.

LTP at CA3–CA1 synapses is believed to depend on NMDAR activation [38,39]. To evaluate the maintenance of the NMDAR-dependent plasticity mechanism, we applied the uncompetitive antagonist MK-801 (10 μM). In the control group, MK-801 application almost completely prevented LTP induction by both TBS (Figure 5a,c; 1.15 ± 0.06; N = 6 rats, *n* = 10 slices) and HFS protocols (Figure 5b,d; 1.07 ± 0.06; N = 8 rats, *n* = 9 slices). In the experimental groups, the effect of MK-801 was similar. No significant LTP was induced in the presence of MK-801 (TBS: 1.08 ± 0.07; N = 5, *n* = 7; HFS: 1.12 ± 0.08; N = 9, *n* = 10). Thus, our results confirm that LTP induction was an NMDAR-dependent process in both control and FS animals.

**Figure 6:**
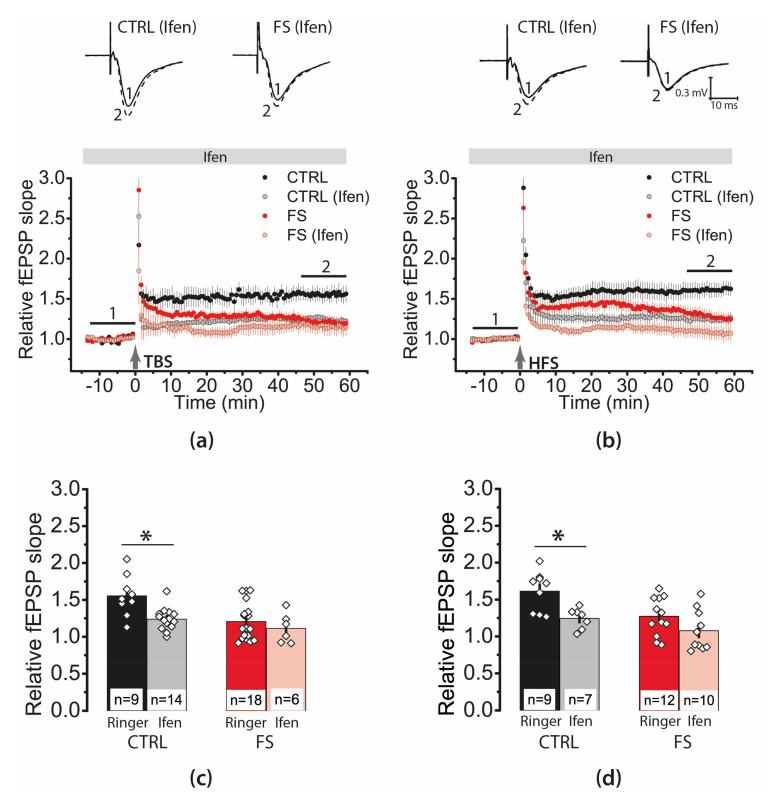
GluN2B-containing NMDARs were involved in the induction of LTP in both control and post-FS animals. The relative fEPSP slope in the control and experimental groups in the presence of ifenprodil (Ifen, 3 μM), a selective GluN2B-containing NMDAR antagonist, before and after TBS (**a**) or HFS (**b**). Note that no synaptic plasticity was detected in the presence of ifenprodil in FS groups. (**c**,**d**) Diagrams illustrating the magnitude of plasticity in the control and post-FS groups in the presence of ifenprodil, after TBS (**c**) or HFS (**d**). Note that no synaptic plasticity was detected in the presence of ifenprodil in FS groups. Two-way ANOVA following Tukey post hoc tests were used. * *p* < 0.05.

Synaptic plasticity properties depend on the subunit composition of NMDARs [40]. To assess the impact of GluN2B-containing NMDARs, we used their selective antagonist ifenprodil (3 μM). We found that ifenprodil significantly affected synaptic plasticity both with the TBS protocol (Figure 6a,c; two-way ANOVA, ifenprodil (+/−): F_1,43_ = 8.0, *p* < 0.01) and the HFS protocol (Figure 6b,d; F_1,36_ = 13, *p* < 0.001). The effect of ifenprodil was similar in the control and experimental groups (TBS protocol: group (control/FS) × ifenprodil (+/−), F_1,43_ = 2.3, *p* = 0.13; HFS protocol: F_1,36_ = 0.74, *p* = 0.39). In control animals, ifenprodil reduced the magnitude of LTP almost two-fold (TBS: 1.24 ± 0.04; N = 9 rats, *n* = 14 slices; Figure 6a,c; HFS: 1.24 ± 0.05; N = 6, *n* = 7; Figure 6b,d). In experimental rats, LTP production was blocked regardless of the protocol used (TBS: 1.11 ± 0.08, N = 6, *n* = 6; Figure 6a,c; HFS: 1.08 ± 0.08; N = 7, *n* = 10; Figure 6b,d).

Thus, GluN2B-containing NMDARs were involved in the induction of LTP in both the control and experimental animals. The complete blockade of LTP in FS rats may be because GluN2B-containing receptors predominate among NMDARs. Another possibility is that ifenprodil further blocks the initially lower current through NMDARs. To determine which of these causes is more feasible, we examined the expression of different glutamate receptor subunits.

### 2.5. The Relative Expression of NMDAR and AMPAR Subunit Genes Does not Change after FS

**Figure 7:**
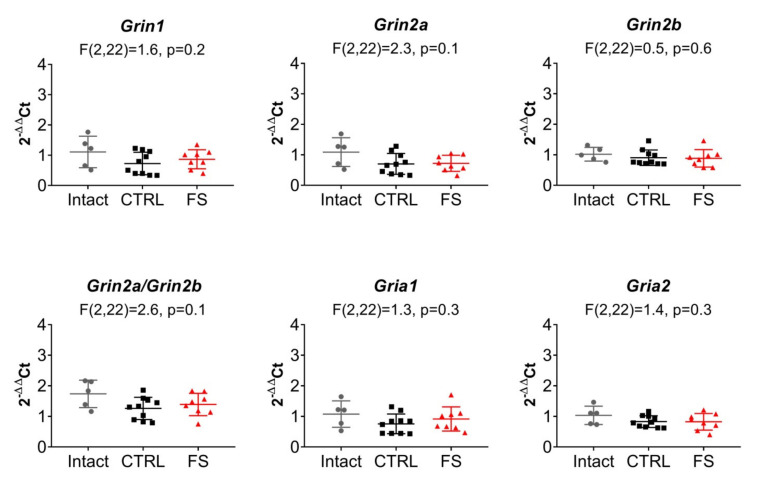
The relative expression of the Grin1, Grin2a, Grin2b, Gria1, Gria2 genes, and Grin2a/Grin2b ratio in the dorsal hippocampal area after febrile seizures. CTRL—control group; FS—experimental group. One-way ANOVA followed by Tukey’s post hoc multiple comparison tests. Data are presented as a mean with a standard error of the mean. Each dot (circle, rhombus, square) represents a value obtained in individual animal.

We compared the expression of NMDAR (*Grin1*, *Grin2a*, *Grin2b*) and AMPAR (*Gria1*, *Gria2*) subunit genes in the dorsal region of the hippocampus in three groups of rats at P21. In addition to the control and FS groups, a group of intact animals was used in this study. No changes in the mRNA production of glutamate receptor subunit genes were found; the *Grin2b/Grin2a* expression ratio was not changed either (Figure 7).

The results suggest that changes in the subunit composition of NMDARs and AMPARs are not likely to occur in this model of febrile seizures. The absence of changes in the expression value of the obligate subunit GluN1 also indicates that the total number of NMDARs is most probably not altered. Thus, the possible reason for the decrease in synaptic plasticity may be due to a dysfunction of NMDARs.

### 2.6. Febrile Seizures Affect the Properties of NMDAR-Mediated Synaptic Currents Evoked by TBS

**Figure 8:**
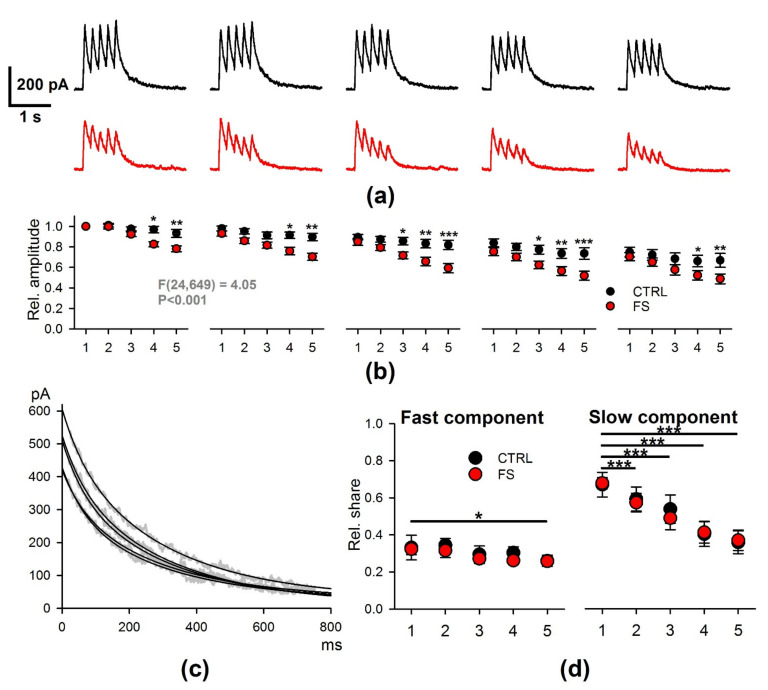
The properties of NMDAR-mediated currents evoked by TBS were disturbed in FS rats. (**a**) The representative voltage-clamp recordings of NMDAR-mediated currents, induced by TBS in CA1 neurons in control (black) and FS rats (red). (**b**) The average amplitudes of the individual peaks of current within the responses, normalized to the amplitude of the first response. Mixed-model ANOVA revealed a significant interaction between the factors (FS × stimulus number). Asterisks indicate the significant difference between the values of control and FS rats for the same stimulus number according to Dunnett’s post hoc test. (**c**) The representative recordings of the 90–10% decays of NDMAR-mediated currents evoked during the TBS. The currents were fitted with the biexponential function with the time constants of $\tau_{fast}$ = 65 ms and $\tau_{slow}$ = 300 ms. (**d**) The relative contribution of the fast and slow components of the response during the TBS. Mixed-model ANOVA did not reveal a difference between the control and FS rats. Asterisks indicate the significant difference with the first response. * *p* < 0.05; ** *p* < 0.01; *** *p* < 0.001.

The decline of LTP after febrile seizures may be due to the weakening NMDAR-dependent calcium current. Therefore, we compared the NMDAR-mediated response during TBS using the whole-cell patch-clamp method. In both control and FS rats, the amplitude of responses decreased during the trains, but the magnitude of this decrease was different (Figure 8a,b). We normalized the amplitudes of the EPSCs to the amplitude of the first peak and performed a mixed-model ANOVA. The analysis revealed that, in the FS group, the amplitude of NMDAR-mediated current peaks decreased in a more pronounced manner within each of the five responses (F_24,649_ = 4.05, *p* < 0.001). Thus, NMDARs exhibited a faster desensitization in FS groups and provided less calcium entry into the postsynaptic terminals during the induction protocol. In turn, this may be the reason for the impaired production of LTP.

Since the kinetics of NMDAR desensitization strongly depends on their subunit composition [41], we additionally tested, using an electrophysiological approach, whether the subunit composition of NMDARs changed after FS. The decay phase of the NMDAR-mediated eEPSC depends on the type of GluN2 subunits and, in pyramidal neurons, it can be fitted with a double exponential function with the fast and slow time constants. Previously, we have shown that the GluN2B-containing NMDAR antagonist ifenprodil selectively blocks the component with the slow time constant [42]. We investigated whether the relative contribution of these components is affected by FS and whether the relative contribution of fast and slow components is affected by TBS differently in control and FS rats.

We confirmed that, in control and FS rats, the decays of the current fitted by two exponential functions with the time constants $\tau_{fast}$ = 65 ms and $\tau_{slow}$ = 300 ms (Figure 8c), and the relative contribution of the fast and slow components, did not differ between FS and control groups for the first response. Next, using the mixed-model ANOVA, we revealed that the relative contribution of slow components decreased during the TBS (F_4,109_ = 56.3, *p* < 0.001). Still, no effect of FS (F_1,109_ = 0.01, *p* = 0.9), as well as no significant interactions between the response number and FS, were detected (F_4,109_ = 0.6, *p* = 0.7). Similar results were found for the fast decaying component (stimulus number: F_4,109_ = 3.5, *p* = 0.011; FS: F_1,109_ = 0.27, *p* = 0.6; stimulus number × FS: F_4,109_ = 0.23, *p* = 0.9).

Together with qRT-PCR data, these results suggest that FSs do not affect the subunit composition of NMDARs.

### 2.7. D-Serine Partially Restore LTP in the FS-Rats

The activation of NMDARs requires glutamate and the co-agonist glycine or D-serine in micromolar concentrations. The binding of glutamate to GluN2 reduces the affinity of GluN1 for glycine/D-serine through negative allosteric modulation; this leads to a gradual decrease in ionic current through NMDAR in the presence of agonists.

NMDAR desensitization is manifest with submicromolar glycine and decreases with higher concentrations of glycine. This is referred to as the glycine-dependent desensitization [41,43,44]. Hippocampal astrocytes retain the ability to control LTP involving Ca^2+^-dependent D-serine release [45], and the astrocyte–neuron relationships are disturbed in epilepsy [46]. Recently, we showed that the application of D-serine fully restored the initial phase of LTP (5–15 min) in the hippocampus of the rat with a lithium–pilocarpine model of the temporal lobe epilepsy [47]. Therefore, our next set of experiments was designed to evaluate the effects of D-serine (10 μM) application.

**Figure 9:**
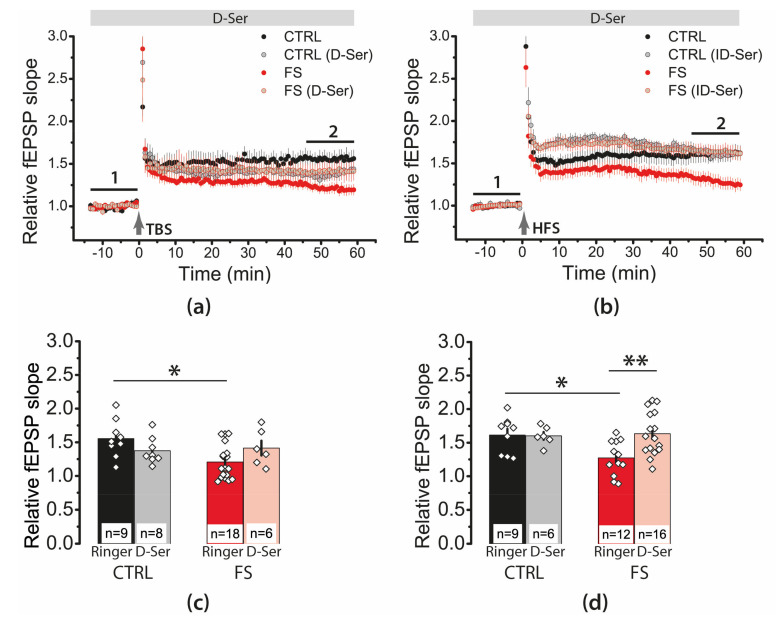
D-serine, a co-agonist of NMDARs, enhanced LTP in the FS but not control group. The normalized fEPSP slope in the control and FS groups in the presence of the NMDAR co-agonist D-serine (10 μM), before and after TBS (**a**) or HFS (**b**). (**c**,**d**) Diagrams illustrating the magnitude of plasticity in the control and experimental groups in the presence of D-serine after TBS (**c**) or HFS (**d**). Two-way ANOVA following Tukey post hoc tests: * *p* < 0.05, ** *p* < 0.01.

We found that D-serine affects synaptic plasticity differently in control animals and FS rats (Figure 9), both when using the TBS protocol (two-way ANOVA, group (FS/control) × D-serine (+/−), F_1,37_ = 5.1, *p* < 0.01) and HFS (F_1,39_ = 4.3, *p* < 0.05). In FS rats, the application of D-serine restored the LTP magnitude to control values. In the case of the HFS protocol, D-serine application increased the LTP magnitude from 1.27 ± 0.07 (N = 6 rats, *n* = 12 slices) to 1.63 ± 0.08 (N = 8 rats, *n* = 16, *p* < 0.01) in FS rats. Our findings suggest that the neuron–glial relationships may be impaired after FS.

### 2.8. The Simulation of the NMDAR-Mediated Response to TBS

**Figure 10:**
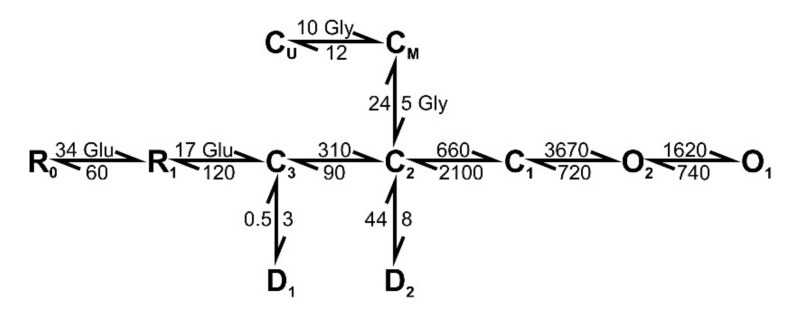
The kinetic scheme used for simulating the gating mechanisms of NMDARs. Rate constants above each arrow are given in s^−1^, except for the glycine-binding and glutamate-binding rate constants, which are in µM^−1^s^−1^. Gly and Glu indicate the glycine and glutamate concentrations (in µM), respectively.

To examine whether the decrease in ambient glycine/D-serine concentration can affect NMDAR desensitization during the TBS, we implemented a mathematical model of NMDAR kinetics (Figure 10).

**Figure 11:**
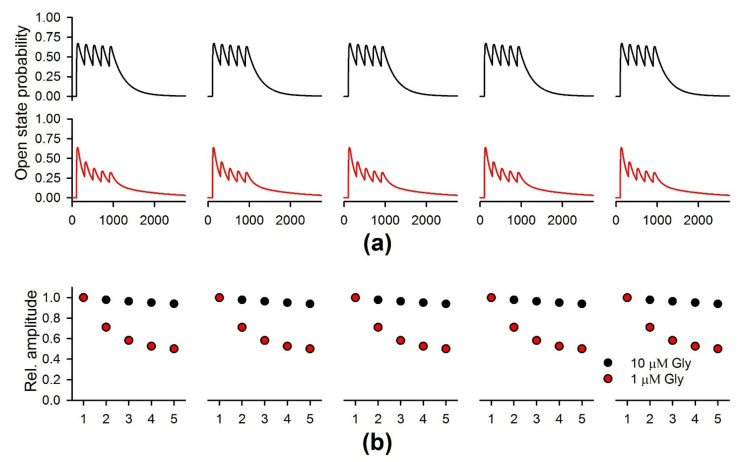
The simulations of the NMDAR-mediated response to TBS. (**a**) The sums of the open state probability (O1 + O1), which reflect the time course of the NMDAR-mediated synaptic currents induced by TBS. The upper traces (black) represent the 5 responses, simulated with 10 µM of ambient glycine. The lower traces (red) were simulated with 1 µM of ambient glycine. (**b**) The amplitudes of the individual peaks of open state probability within the responses normalized to the first peak amplitude of the first response.

The simulations were performed with two concentrations of ambient agonist: 10 µM and 1 µM. With both concentrations of glycine, the implementation of TBS induced the NMDAR responses, which resembled the ones observed in the experiment. Each burst caused a single peak of open state probability, resulting in five responses, each having five peaks (Figure 11). When the higher concentration of glycine was present, the individual peaks displayed a slight decrease within each set of five bursts. In the low concentration of glycine, the amplitude of the probability peaks showed a more substantial reduction during the train (Figure 11), comparable to the experimental observations of NMDAR currents during the TBS. Thus, the simulation demonstrates that glycine in low concentration promotes the use-dependent downregulation of NMDAR during the TBS, similar to that observed in the experiments.

## 3. Discussion

Considerable evidence has accumulated that suggests prolonged or repeated neonatal seizures can lead to developing acquired epilepsy and cognitive deficits later in life [16,28,31,48,49]. The exact mechanisms of seizure-induced epileptogenesis and cognitive abnormalities are not yet fully understood, though it is considered that alterations of the excitatory synaptic transmission properties contribute to this process. Due to the high prevalence of seizures in children, understanding the mechanisms of their effects on the immature brain is one of the most critical tasks.

This study used an animal model closely replicating prolonged FSs [3]; to achieve this, we monitored the duration of FSs in animals and included only rats with seizures lasting 15–20 min. The major findings of this study were that febrile seizures in P10 cause a significant attenuation of LTP induction in the hippocampus in rats at P21. This weakening of the hippocampal synaptic plasticity was accompanied by the attenuation of synaptic transmission efficacy in the CA1 area and a slight reduction in the number of neurons in the CA1 and the hilus areas. We suggest that the weakening plasticity induction in FS rats was related to the more pronounced desensitization of NMDARs. The increased desensitization may be due to an insufficient activation of the glycine site of NMDARs, as the glycine site co-agonist D-serine application allows the restoration of synaptic plasticity to a control value.

In this study, we hypothesized that impaired glutamatergic transmission in the FS model might be primarily related to the disruption in properties of AMPARs and NMDARs, because such disorders have been shown in various models of neonatal seizures. For example, early life hypoxia-induced seizures lead to the enhancement of AMPAR-mediated signaling in CA1, associated with increased receptor phosphorylation [50]. The enhanced tyrosine phosphorylation of NMDAR subunits was also observed following neonatal hypoxia–ischemia [51]. In the model of hypoxic neonatal seizures, an increase in GluN2A phosphorylation in the hippocampus was shown [52]. The hypoxia-induced seizures may also result in the enhanced expression of GluA2-lacking Ca^2+^-permeable AMPA receptors in the hippocampus [31,53]. The most regularly reported disturbances at the early stages of epileptogenesis include an increase in the relative contribution of GluN2B-containing NMDARs, which was shown in pilocarpine and lithium-pilocarpine models [42,54,55]. Similar results were obtained in the pentylenetetrazole-induced kindling [56].

The effects of FSs on the glutamatergic system are currently less investigated. Early life frequently repetitive FSs resulted in selective deficits in GluN2A subunit tyrosine phosphorylation after NMDA treatment in the hippocampal CA1 areas of adult rats [24]. Chen et al. showed that GluN2B Tyr1472 phosphorylation gradually increased and maintained a high level for 7 days after prolonged FSs, without any apparent change in the total GluN2B expression level [57].

In the present study, we did not observe any changes in mRNA expression levels of AMPARs or NMDARs subunits. Although the expression levels of receptor subunits at the protein and mRNA levels may not coincide, the decays of NMDAR-mediated currents were similar in control and FS groups, suggesting that the subunit composition of NMDARs was not affected by FSs. However, the functional activity of NMDARs was altered in the FS group: NMDARs exhibited a faster desensitization in FS groups compared with controls.

NMDAR desensitization results from several different concurrent processes. After NMDAR activation, at least two different types of desensitization occur [41]. (1) Glutamate binding to GluN2 reduces the affinity of GluN1 to glycine via negative allosteric modulation; this results in the gradual reduction in the ion current through the NMDAR in the presence of agonists [43]. An increase in glycine concentration abolishes this effect [58]. (2) Calcium-dependent desensitization results from calcium binding to the intracellular portion of the GluN1 subunit after its entrance to the cell [41]. Both types of desensitization depend on the NMDAR subunit composition [41].

Since we did not detect any changes in the NMDAR subunit composition using a PCR analysis, we suppose that the changes may be related to the availability of glycine site agonists. The following data support this assumption. Extracellular glycine concentrations vary with brain region and neuronal activity. In the hippocampus, the synaptic availability of glycine is mainly under the control of glycine transporter 1 (GlyT1) expressed in astrocytes and postsynaptic neurons [59,60]. Shen et al. (2015), using two different rodent models of epilepsy, demonstrated a robust overexpression of GlyT1 in the hippocampal formation, suggesting dysfunctional glycine signaling in epilepsy [61]. It should be noted that changes in the hippocampal glycine receptor expression have also been reported in patients with temporal lobe epilepsy, suggesting a dysregulation of glycinergic signaling in epilepsy [62]. Thus, we can assume a decrease in the concentration of available glycine after FSs and, consequently, an increase in the desensitization of NMDRs. This assumption is supported by the fact that the addition of D-serine, a glycine site agonist, restored LTP in our experiments.

Another possible cause of increased NMDAR desensitization and impaired synaptic plasticity could be disturbances in neuron–glial relationships. Hippocampal astrocytes retain the ability to control LTP within or near their individual territories, involving the Ca^2+^-dependent D-serine release [45]. Thus, astrocytes can regulate the local D-serine supply and might be able to deliver D-serine to specific NMDAR populations, thereby decreasing their desensitization. Previously, we showed that the application of D-serine fully restored the initial phase of LTP in hippocampal slices of pilocarpine-treated rats [47]. In this study, the effect of D-serine was more pronounced, suggesting that the hypothesis of the impaired neuron–glia interactions should be further explored.

Experimentally induced FSs cause a robust inhibition of interastrocytic gap junctional coupling [63], which may affect the excitability of hippocampal neurons after FS and facilitate the development of epilepsy [20,22]. In the repetitive FS models, both long-term astrocyte activation [64] and pronounced ultrastructural changes in the astroglia of the hippocampus and temporal lobe neocortex were shown. The damage of granular endoplasmic reticulum and mitochondria was the primary manifestation [65], leading to disorders in some intercellular biochemical events, such as an abnormal protein synthesis or the inhibition of oxidative phosphorylation.

In the present study, we also confirmed that early life FSs do not lead to significant neuronal cell death, supporting the previous studies showing that the immature hippocampus is resistant to seizure-induced neuronal death [20,66]. However, the present study demonstrates a 10% neuronal loss in the pyramidal layer of the hippocampal CA1 area and hilus, but not in CA3 or DG. Furthermore, the distribution of affected neurons was partially similar to that reported in a previous study [19], in which the authors, using the silver-staining method, identified the most damaging significant and prolonged alterations in the physicochemical properties of neurons in the pyramidal layer of the hippocampal CA1 and all of the CA3 subfields.

Thus, our study suggests that early life FSs do not result directly in hippocampal cell death, but lead to multiple functional disturbances in glutamatergic neurotransmission, including alterations the in synaptic plasticity. These disturbances could eventually lead to an epileptic state and be the primary mechanism of cognitive dysfunctions.

## 4. Materials and Methods

### 4.1. Animals and FS Model

All the experiments were approved by the Sechenov Institute of Evolutionary Physiology and Biochemistry Ethics Committee and carried out following local guidelines on the treatment of laboratory animals. These guidelines fully comply with Russian and international standards for animal studies. Female Wistar rats, together with their litters, were kept under standard conditions at room temperature, with free access to water and food. Only 10 pups were abandoned in the litter. FS was induced on P10. Pups were placed on the bottom of a 10 L glass chamber. The temperature was maintained at 46 °C. The animal temperature was measured rectally [67].

Before the experiment, the temperature was 31.1 ± 0.1 °C, and at the beginning of the seizure, it was 39.8 ± 0.1 °C. After the onset of seizures, the temperature was measured every 2 min. If the temperature raised above 41 °C, the pups were moved to a cool surface for 2 min and then returned to the chamber. Hyperthermic animal temperatures (39–41 °C) were maintained for 25 min.

The behavioral seizures in this paradigm were stereotyped, consisting of the arrest of heat-induced hyperkinesis followed by facial automatisms, often accompanied by body flexion, followed by myoclonic twitches and clonic seizures. After hyperthermia, animals were placed on a cold surface until the core temperature returned to the normal range and then returned to the home cage. After hyperthermia, the pups’ weight changed insignificantly (<3% change in body weight), indicating only slight dehydration symptoms. The mortality rate at hyperthermia and during the following 30 min was less than 1%. Only animals with FS that lasted at least 15 min were included in the study (N = 43).

Littermates used for controls were taken from the cage for the same time but maintained at room temperature (N = 42). Additionally, a group of intact animals (N = 5) was used to study the expression of glutamate receptor subunits. Rats from the intact group were not exposed to any treatments.

### 4.2. Brain Slices Preparation

Hippocampal slices were prepared as previously described [68]. Briefly, at P21–23, the rats were decapitated. The brain was rapidly removed. Horizontal brain slices (400 μm) were cut using a vibratome HM 650 V (Microm, Walldorf, Germany) in chilled artificial cerebrospinal fluid (ACSF; t = 0 °C), which was aerated with carbogen (95% O_2_ and 5% CO_2_). Composition of ACSF (in mM): 126 NaCl, 2.5 KCl, 1.25 NaH_2_PO_4_, 1 MgSO_4_, 2 CaCl_2_, 24 NaHCO_3_, 10 glucose. The slices were then incubated at 35 °C for 1 h and then left at room temperature. After incubation, the slices were transferred to a recording chamber where field responses were registered at 25–27 °C. In the recording chamber, the ACSF flow rate was 5–7 mL/min.

### 4.3. Field Potential Recordings

Extracellular field excitatory postsynaptic potentials (fEPSPs) were recorded from the CA1 stratum radiatum of the hippocampus with glass microelectrodes (0.2–1.0 MΩ). Each slice was stimulated with increasing amplitude currents (25 to 300 μA, 25 μA step), and the amplitude, 20–80% rising phase slope, and fiber volley amplitude (FVs) were measured for each fEPSP. The efficacy of neurotransmission was determined with a sigmoidal Gompertz function as described previously [36]. The stimulation current amplitude for the LTP experiment was 40–50% of the current intensity, inducing population spikes. Stimuli were delivered every 20 s via an A365 stimulus isolator (World Precision Instruments, Sarasota, FL, USA). Responses were amplified by a Model 1800 amplifier (A-M Systems, Carlsborg, WA, USA), then digitized with ADC/DAC NI USB-6211 (National Instruments, Austin, TX, USA) using WinWCP v5.x.x software (University of Strathclyde, Glasgow, UK). The recordings were analyzed using Clampfit 10.2 software (Axon Instruments, San Jose, CA, USA).

Two types of stimulation protocols were used for LTP induction. (1) TBS: five bursts of five pulses with a frequency of 100 Hz, with an interval of 200 ms between bursts. The stimulation was repeated five times every 10 s. (2) HFS: 3 trains consisting of 100 pulses at 100 Hz applied every 20 s.

A 20 min baseline period preceded LTP induction. Potentiated fEPSPs were recorded for 60 min following stimulation. LTP was quantified by calculating the ratio of the average slope of the potentiated fEPSPs (50–60 min after stimulation) and the baseline ones (10 min before stimulation). MK-801 (10 μM), an uncompetitive NMDAR antagonist, ifenprodil (3 μM), a GluN2B subunit-selective NMDAR antagonist, D-serine, a co-agonist of NMDARs were obtained from Sigma (St. Louis, MO, USA). These drugs were diluted in distilled water and bath applied.

The paired-pulse ratio (PPR) was calculated as the second to the first fEPSP amplitude ratio.

### 4.4. The Whole-Cell Patch-Clamp Recordings

The pyramidal neurons were visualized using a Zeiss Axioskop 2 microscope (Zeiss, Oberkochen, Germany), equipped with differential interference contrast optics and a video camera (Grasshopper 3 GS3-U3-23S6M-C; FLIR Integrated Imaging Solutions Inc., Wilsonville, OR, USA). Patch electrodes (3–5 MΩ) were pulled from borosilicate glass capillaries (Sutter Instrument, Novato, CA, USA) using a P-1000 pipette puller (Sutter Instrument, Novato, CA, USA). A cesium methanesulfonate-based pipette solution (composition in mM: 127 CsMeSO3, 10 NaCl, 5 EGTA, 10 HEPES, 6 QX314, 4 ATP-Mg, and 0.3 GTP; pH adjusted to 7.25 with CsOH) was used for voltage-clamp recordings. Whole-cell recordings were performed using a MultiClamp 700B (Molecular Devices, Sunnyvale, CA, USA) patch-clamp amplifier and an NI USB-6343 A/D converter (National Instruments, Austin, TX, USA) using WinWCP 5 software (University of Strathclyde, Glasgow, UK). The data were filtered at 10 kHz and sampled at 20 kHz. In all cells included in the sample, access resistance was less than 20 MΩ and remained stable (≤20% increase) across the experiment. The liquid junction potential was compensated offline for the voltage-clamp recordings by subtracting 7 mV. A bipolar stimulating electrode was placed in the same area as for the field-potential recordings. NMDAR-mediated eEPSCs were recorded at +40 mV in the presence of gabazine (10 µM, Alomone Labs, Jerusalem, Israel) and DNQX (10 µM, Tocris Bioscience, Bristol, UK).

Changes to the time course of the NMDAR-mediated current during the TBS were described using the non-linear regression analysis on the decay phase (90–10%) [42]. We utilized the biexponential function:

(1) $$I\left(t;A_{fast},\tau_{fast},A_{slow},\tau_{slow}\right)=A_{fast}*\exp\left(-\frac{t}{\tau_{fast}}\right)+A_{slow}*\exp\left(-\frac{t}{\tau_{slow}}\right)$$

where $A_{fast}$ and $\tau_{fast}$ are the amplitude and time constant of the fast decaying component; $A_{slow}$ and $\tau_{slow}$ are the amplitude and time constant of the slow decaying component.

For each of five NMDAR-mediated currents induced by TBS, the relative shares of the fast and slow decaying component were calculated as follows:

(2) $$Rel.share_{fast}=\frac{A_{fast}}{A_{fast}1+A_{slow}1}$$

(3) $$Rel.share_{slow}=\frac{A_{slow}}{A_{fast}1+A_{slow}1}$$

where $A_{fast}1$ and $A_{slow}1$ are the amplitudes of the fast and slow decaying components of the first out of five responses, respectively.

### 4.5. Quantitative PCR (qRT-PCR)

Rats were decapitated at P21. Brains were quickly removed, frozen, and stored at −80 °C until dissection. The dorsal hippocampus was dissected using a cryostat OTF5000 (Bright Instrument, Huntingdon, UK) according to the rat brain atlas [69]. Total RNA was isolated using the ExtractRNA reagent (Evrogen, Moscow, Russia) following the manufacturer’s protocol. RNA concentration was assessed spectrophotometrically using NanoDrop™ Lite (Thermo Fisher Scientific, Wilmington, DE, USA).

Total RNA (2 μg), oligo-dT-primers, and 9-mer random primers (0.5 µg per 1 µg RNA and 0.25 µg per 1 µg RNA, respectively) (DNA Synthesis Ltd., Moscow, Russia) and MMLV reverse transcriptase (100 units per 1 µg RNA; Evrogen, Moscow, Russia) were used for reverse transcription. The reaction was carried out in a total volume of 25 µL. We mixed primers and 8 µL of RNA solution and incubated 10 min at 70 °C and quickly cooled to 4 °C for primer annealing. Then, we added reverse transcriptase-containing reaction mix, and samples were incubated 1 h at 42 °C and 10 min at 65 °C. After this step, all samples were 7-fold diluted. qPCR was performed in a total volume of 10 µL with 0.8 µL cDNA, 0.75 units of TaqM-polymerase (Alkor Bio, St. Petersburg, Russia), 3.5 mM Mg^2+^, specific forward, reverse primers and hydrolysis (TaqMan) probes (see Table 1). Nucleotides were synthesized by DNA Synthesis Ltd. (Moscow, Russia). PCR reactions were performed in a C1000 Touch thermal cycler combined with a CFX96 Touch™ Real-Time PCR Detection System (Bio-Rad, Hercules, CA, USA) in triplets, simultaneously with no template and no reverse transcription control samples. The relative expression of *Grin1*, *Grin2a*, *Grin2b*, *Gria1* and *Gria2* genes was calculated using the 2^−ΔΔCt^ method [70], normalized against the relative expression of *Ppia* gene, which is stable in the seizure models [71].

### 4.6. Histology

At P21–23, rats were deeply anesthetized with a Zoletil/Xylazine mixture (Virbac, France) and sacrificed by transcardial perfusion with ice-cold 0.01 M PBS (pH = 7.4) and next with ice-cold 4% paraformaldehyde (PFA) in 0.1M PB (pH = 7.4) at a rate of 10 mL/min. Then, the brains were removed and fixed in 4% PFA at 4 °C for 24 hs. The brains were rinsed from PFA and cryoprotected in 30% sucrose in PBS at 4 °C for 2–3 days. Finally, the brains were frozen in cooled (<−50 °C) isopentane (78-78-4, Isopentane Solution, Sigma-Aldrich, St. Louis, MO, USA) and stored at −80 °C until cutting. An amount of 20 μm thick coronal serial sections were cut with a Bright OTF5000 cryostat (Bright Instrument Co Ltd., Huntingdon, UK) from 2.6 to 3.6 mm caudal to bregma, mounted on slides with adhesive coating Super Frost Plus (J1800AMNZ, Fisher Scientific UK Ltd., Loughborough, UK). Once dried for one day, the unstained slides were treated in a series of solutions for Nissl staining. Sections were defatted in a mixture of ethanol (EtOH) and chloroform (Ekos-1, Moscow, Russia) (1:1) for 1–3 h, and then rehydrated in EtOH solutions of decreasing concentration (3 steps in 96% EtOH and 2 steps in 70% EtOH; 2 min each step), followed with 2 rinses in distilled water (1 min each), followed by 5 min in 0.05% thionin solution (pH 4.5). Sections were then rinsed in distilled water (2 steps, 1 min each) and dehydrated in increasing concentrations of ethanol solutions. Finally, sections were cleared in microclearing (Diapath, Martinengo, Italy; 2 steps of 15 and 20 min) and then coverslipped with VitroGel (Ergo Production, Moscow, Russia). 

The Nissl stained sections were analyzed using Leica Microscope AF 7000 (Leica Microsystems, Wetzlar, Germany) under ×400 magnification. For morphological analysis, neuronal counts were performed on every 5th section (yielding 8–10 sections from one rat hippocampus). The distance between the analyzed sections was 100 µm. The number of neurons in digital micrographs was counted per 100 μm for the cell layer in CA1, CA3, hilus and dentate gyrus using ImageJ (U. S. National Institutes of Health, Bethesda, MD, USA).

### 4.7. Simulations

A kinetic model of NMDARs proposed by [58] with glutamate-binding steps appended as described in [76] was used for simulation of TBS-induced NMDAR-mediated currents. This kinetic scheme (Figure 1) included two glutamate binding steps (R_0_ and R_1_), three closed states (C_3_, C_2,_ and C_1_), two desensitized states (D_1_ and D_2_), two open states (O_1_ and O_2_), and two glycine-binding steps (C_U_ and C_M_). The synaptic input during the TBS was simulated as the 1 ms pulses of 1 mM glutamate (5 sets of 5 pulses, repeated every 100 s). The concentration of glycine was set equal to a constant value. The corresponding system of the rate equations (eleven first-order differential equations, one per each state of probability) was solved numerically using the Wolfram Mathematica 12 (Wolfram Research, Champaign, IL, USA). As the two open stats of NMDAR have similar conductance [58,77], the time course of the macroscopic current was calculated as the sum of the solutions for the two conducting states.

### 4.8. Statistical Analysis

The statistical analysis and graphical representation of the results were performed using OriginPro 8 (OriginLab Corporation, Northampton, MA, USA and SigmaPlot 12.5 (Systat Software Inc., Palo Alto, CA, USA). Dixon’s Q test (at a 90% confidence level) was used to identify and reject outliers. The normality of the sample data was evaluated using the Kolmogorov–Smirnov test. The equality of variance was assessed using the Levene median test. Statistical significance was assessed using Student’s *t*-test and ANOVA as stated in the text. All data are presented as mean ± standard error of the mean. *p* < 0.05 was considered statistically significant.

## Figures and Tables

**Table 1:** Primers and probes used in qRT-PCR.

Gene Symbol RefSeq Accession Number	Nucleotide Sequences (Forward, Reverse, TaqMan-Probe)	Reference
*Ppia* NM_017101	AGGATTCATGTGCCAGGGTG CTCAGTCTTGGCAGTGCAGA ROX-CACGCCATAATGGCACTGGTGGCA-BHQ1	[72]
*Grin1* NM_017010	GTTCTTCCGCTCAGGCTTTG AGGGAAACGTTCTGCTTCCA FAM-CGGCATGCGCAAGGACAGCC-BHQ1	[73]
*Grin2a* NM_012573	GCTACACACCCTGCACCAATT CACCTGGTAACCTTCCTCAGTGA FAM-TGGTCAATGTGACTTGGGATGGCAA-BHQ1	[74]
*Grin2b* NM_012574	CCCAACATGCTCTCTCCCTTAA CAGCTAGTCGGCTCTCTTGGTT FAM-GACGCCAAACCTCTAGGCGGACAG-BHQ1	[74]
*Gria1* NM_031608	TCAGAACGCCTCAACGCC TGTAGTGGTACCCGATGCCA ROX-TCCTGGGCCAGATCGTGAAGCTAGAAAA-BHQ1	[75]
*Gria2* NM_017261	CAGTGCATTTCGGGTAGGGA TGCGAAACTGTTGGCTACCT FAM-TCGGAGTTCAGACTGACACCCCA-BHQ1	[75]

## Data Availability

The data presented in this study are available on request from the corresponding author.
